# Anal HPV infection and correlates in HIV-infected patients attending a Sexually Transmitted Infection clinic in Brazil

**DOI:** 10.1371/journal.pone.0199058

**Published:** 2018-07-05

**Authors:** Neide Aparecida Tosato Boldrini, Lays Paula Bondi Volpini, Luciana Bueno de Freitas, Carlos Musso, Paulo Roberto Merçon de Vargas, Liliana Cruz Spano, Angelica Espinosa Miranda

**Affiliations:** 1 Post-Graduation Program in Infectious Diseases, Federal University of Espírito Santo, Vitoria, Brazil; 2 Department of Gynecology and Obstetrics, Center of Health Sciences, Federal University of Espírito Santo, Vitória, Brazil; 3 Department of Pathology, Center for Health Sciences, Federal University of Espírito Santo, Vitória, Brazil; Istituto Nazionale Tumori IRCCS Fondazione Pascale, ITALY

## Abstract

**Objective:**

To estimate the prevalence of anal HPV infection, genotype distribution, intraepithelial neoplasia (AIN) and correlates in a cohort of HIV-infected patients attending at Sexually Transmitted Infections (STI) clinic in Brazil.

**Study design:**

A descriptive analysis was performed which includes, demographic, behavioral and clinical data. Anal specimens from HIV-positive men and women were collected during a regular visit and they were used for cytology and histopathology tests, as well as for HPV molecular identification.

**Results:**

A total of 223 patients (143 females and 80 males) were enrolled in the study and, HPV was identified in 68.6% of the sample. The frequency of HR-HPV, HPV16/18 and multiple HPV infection were similar in both groups. The upstream regulatory region (URR) sequencing was carried out in 38 samples identified as HPV16-positive, and European variants were the most frequent (69.2%), followed by Africans (25.6%) and Asiatic-Americans (5.1%). Having more than 20 sexual partners was associated with multiple HPV infection (p = 0.000) while, anal sex and the first intercourse before 15 years of age was a risk factor for any HPV infection (p = 0.001). Being MSM (men who have sex with men) was a risk factor for any HPV and multiple infections (p = 0.002). The CD4 count >500 cells/mm^3^ was a protective factor for the HPV16/18 (p = 0.048) and multiple infections (p = 0.023), and the undetectable viral load and HAART treatment were both protective for any HPV (p = 0.010), HR-HPV (p = 0.091) and multiple infections (p = 0.006). Abnormal anoscopy was found in 23.7% (53/223) of the total number of patients, and this was significantly associated with all types of investigated HPV infections (p<0.0001).

**Conclusions:**

In this study, anal HPV infection was common among young HIV-positive men and women, particularly in MSM. Anal cancer screening in patients at risk, such as those who are HIV-positive, and mainly those with anal HPV infection and a history of STI, will increase the likelihood of detecting anal intraepithelial neoplasia.

## Introduction

Human papillomavirus (HPV) is a common sexually transmitted infection (STI)that can be categorized into two groups, low risk (LR-HPV) and high risk (HR-HPV) with respect to their risk of progression to malignancy [1]. Latent papillomaviruses are detectable only through the demonstration of HPV DNA in clinically and histologically normal skin and mucosa [2]. Most sexually active individuals will acquire at least one genotype of anogenital HPV infection at some time during their lifetime [3], but there are some risk and behavioral factors that could increase the infection frequency or persistence of the virus [4]. HR-HPV types are more disposed to progress to malign lesions, and some of them have variants that differ in biological and epidemiological patterns [5]. Furthermore, several co-infections, such as multiple HR-HPV or HIV co-infection could have a role in the higher probability of the progression of lesions [6].

Several studies identified a fluctuating HPV prevalence around the world, which could be due to some population characteristics, such as age and behaviors or to the sensitivity of the HPV DNA tests [7,8,9]. HR-HPV is associated with 100% of the cervical cancer cases and more than 80% of anal cancer [10,11,12]. The rate of anal cancer has been increasing over the years in patients at higher risk for persistence of HPV virus such as women with previous cervical lesions and in patients with some grade of immunosuppression, as transplanted and HIV positive [13,14,15,16].

The role of HPV in cervical lesions is well documented, but data of anal intraepithelial neoplasia is gaining importance more recently, in both men and women. In men, HPV infection has been strongly associated with anal cancer with approximately 88% of the anal squamous cell cancers occurring annually worldwide [17]. The prevalence of anal HPV infection is higher than cervical HPV infection in women; the same occurs for anal intraepithelial neoplasia (AIN), where the reported prevalence is 23–86% in HIV-positive and 5–22% in HIV-negative women [4,17,18]. Similarities in tumor biology have been demonstrated by anal and cervical cancer. Thus the implementation of an anal cancer screening program could bring a similar success as the cervical cancer program [19,20]. To date, there are no uniform screening guidelines for anal cancer, but some tools could be used in populations at higher risk for anal cancer development, such as a digital anorectal exam, anal Pap cytology and high-resolution anoscopy (HRA) [21].

The relationship between HIV and malignancies has been described, being significantly associated with anal cancer when comparing the general population [10]. Although there are no randomized experiments that demonstrate the effectiveness of this strategy, the anal cancer screening has been discussed and encouraged in groups that are considered to be at risk [22], however, this procedure is not regularly performed during a clinical routine in Brazil. As the higher prevalence and higher risk of complications of HPV infection in HIV-infected patients are well known [23], the screening would be an important preventative tool. Consequently, the goal of this study was to estimate the prevalence of anal HPV infection, HPV genotype distribution and correlates with anal HPV infection and anal intraepithelial neoplasia in HIV-seropositive patients of both genders.

## Materials and methods

This is a cross-sectional study of HIV-positive men and women (18–69 years of age) attending a public STI/HIV screening and treatment center in Vitória, Brazil, from March 2013 to February 2016. The Ethics Committee of Universidade Federal do Espírito Santo (UFES), Brazil approved this study. Participants signed a written informed consent before being included in the study. They were invited to answer an interview about socio-demographic, behavioral and clinical data and performing an anal examination, including sample collection.

### Interview data

A 20-minutes face-to-face interview was conducted with the use of a standardized questionnaire. Enrolled patients answered the interview questions, which included demographic (age, schooling, marital status, family income); behavioral (tobacco use, alcohol and illicit drug use, age at first sexual intercourse, number of sex partners, types of sexual activity, frequency of condom use in the last year and STI history) and clinical data (use of highly active antiretroviral therapy (HAART), CD4 counts and viral load).

### Anal specimen collection

Sample collection was undertaken using a cytobrush inserted 4.0 cm into the anal canal and performing a spiral motion to seize samples from the entire circumference of the anal canal. The samples were spread onto a microscope slide and stained using the Papanicolaou method. The classification of cytological findings was undertaken according to the Bethesda system of cervical cytology [24]. The specimens were fixed in a 10% formalin buffered solution and sent to the laboratories of the Department of Pathology of the University Hospital for processing and analysis. Two pathologists analyzed all the cytological and anal biopsy samples.

### DNA extraction / HPV detection and typing

Anal samples were obtained using cytobrush and placed in 2 ml tube containing TE buffer (10mM Tris-HCl; 1mM EDTA; pH 8.0) and store at -70°C. The DNA was isolated using a QIAamp DNA Mini Kit™ kit (QIAGEN INC, Valencia, California. The USA) according to the manufacturer's instructions. The HPV DNA was detected by amplification with PGMY09/11 primers [25], and the positive samples were genotyped by Restriction Fragment Length Polymorphism (RFLP) [26] and by Reverse Line Blot (RLB) [27]. A fragment of the upstream regulatory region (URR) of all HPV16 positive samples was sequenced and aligned together with HPV16 reference sequences of each sublineage in order to identify the HPV variants [28].

### High-resolution anoscopy (HRA)

Subjects with any grade of anal cytological abnormality or anal HR-HPV were referred for examination using high-resolution anoscopy (HRA). The examination was performed with a colposcope, and a gynecologist with expertise in colposcopy performed all HRA procedures. A cotton ball with 3% acetic acid was used while anal mucosa was observed and the anoscope was moving out of the anal canal. The results of HRA were considered positive or negative for aceto-white lesions, dots, mosaics, atypical vessels following the Consensus terminology of 2016 IANS International Guidelines for Practice Standards in the Detection of Anal Cancer Precursors [29]. Punch anal biopsy was performed on all suspicious areas (visible lesions) after application of acetic acid.

### Statistical analysis

Frequency distribution and descriptive statistics were used to describe the study variables. Estimated prevalence and 95% confidence interval for any HPV type, HR-HPV, HPV16/18 and multiple HPV infection were calculated. Descriptive statistics were calculated in order to describe the most prevalent genotypes in the study. Chi-square analysis or Fisher's exact test were used to evaluate differences in categorical outcome measures. Socio-demographic characteristics were stratified into two groups by gender. Data were coded and stored anonymously in a database. The statistical software SPSS v. 20.0 (Statistical Package for the Social Sciences, IBM, Chicago, USA) was used for the analyses.

## Results

Anal samples were collected from 223 HIV-seropositive patients, being 80 men and 143 women, and the median age was 40.0 years (SD = 11.20). Women and men presented the same average with respect to age, the majority were employed (69%) and had completed a secondary education (57.4%) (Table 1). A total of 64.6% reported previous STI and the most common was the *Condyloma acuminatum* (22.9%), followed by syphilis (16.6%), and 27.7% had more than one STI. The HPV test was positive in 68.6% (153/223) of patients, 71.3% (57/80) among men, and 67.1% (96/143) among women.

**Table 1:** Socio-demographic characteristics of a sample of patients attending an STI clinic in Vitoria, Brazil (n = 223).

Variable	Total (%)	Woman	Man	*p-value*
**Age (years)**	40.6 ±11.2	41.0 ±10.4	40.0 ± 12.6	0.060
**Education level**				< 0.0001
Less than five years	29 (13.9)	24 (17.4)	05 (7.0)	
Primary education	60 (28.7)	47 (34.1)	13 (18.3)	
Secondary education	86 (41.1)	57 (41.3)	29 (40.8)	
Higher education	34 (16.3)	10 (7.2)	24 (33.8)	
**Employed**				0.001
No	48 (22.5)	42 (30.2)	06 (8.1)	
Yes	147 (69.0)	86 (61.9)	61 (82.4)	
Retired	18 (08.5)	11 (07.9)	07 (09.5)	
**Marital status**				< 0.001
Single	87 (41.2)	40 (28.8)	47 (65.3)	
Married	97 (46.0)	76 (54.7)	21 (21.6)	
Separated	13 (06.2)	10 (07.2)	03 (04.2)	
Widow	14 (06.6)	13 (09.4)	01 (07.1)	
**Self-reported STI**				0.015
Yes	144 (67.9)	87 (62.6)	57 (78.1)	
No	68 (32.1)	52 (37.4)	16 (21.9)	
**Body mass index**				0.060
Under weight	21 (10.2)	16 (12.4)	05 (6.5)	
Eutrophic	86 (41.7)	48 (37.2)	38 (49.4)	
Over weight	46 (36.9)	46 (35.7)	30 (39.0)	
Obese	23 (11.2)	19 (14.7)	04 (05.2)	
**HIV diagnosed (years)**				0.003
< 5	93 (41.7)	47 (32.9)	46 (57.5)	
11 to 10	62 (27.8)	43 (30.0)	19 (23.8)	
11 to 20	65 (29.1)	51 (35.7)	14 (17.5)	
> 20	03 (01.3)	02 (01.4)	01 (01.2)	
**Cytology abnormal**	53 (25.5)	26 (19.4)	27 (36.5)	0.007
**AIN (biopsy)**	29 (13.0)	15 (10.5)	14 (17.5)	0.101
**HPV +**	153 (68.6)	96 (67.1)	57 (71.3)	0.315

Regarding cytological abnormalities in anal specimens, ASC-US (Atypical Squamous Cells of Undetermined Significance), LSIL (Low-grade squamous intraepithelial lesion) and HSIL (High-grade squamous intraepithelial lesion) were found in 20.6%, 2.2% and 0.8% of the total samples, respectively. Classifying by gender, abnormal results were significantly more common in males than in females (p = 0.008), and the abnormalities also were also associated with being HPV positive in the male group (p = 0.038). Thirty-three participants were undergoing biopsies with a colposcopic examination of the anal canal. Twenty-nine (18.3%) participants were diagnosed with anal intraepithelial neoplasia (AIN), twenty cases of LSIL, two cases of HSIL and one case of the anal adenocarcinoma. All the cases of HSIL were related to genotypes of HPV 16 or 18 in HIV-positive women.

**Fig 1:**
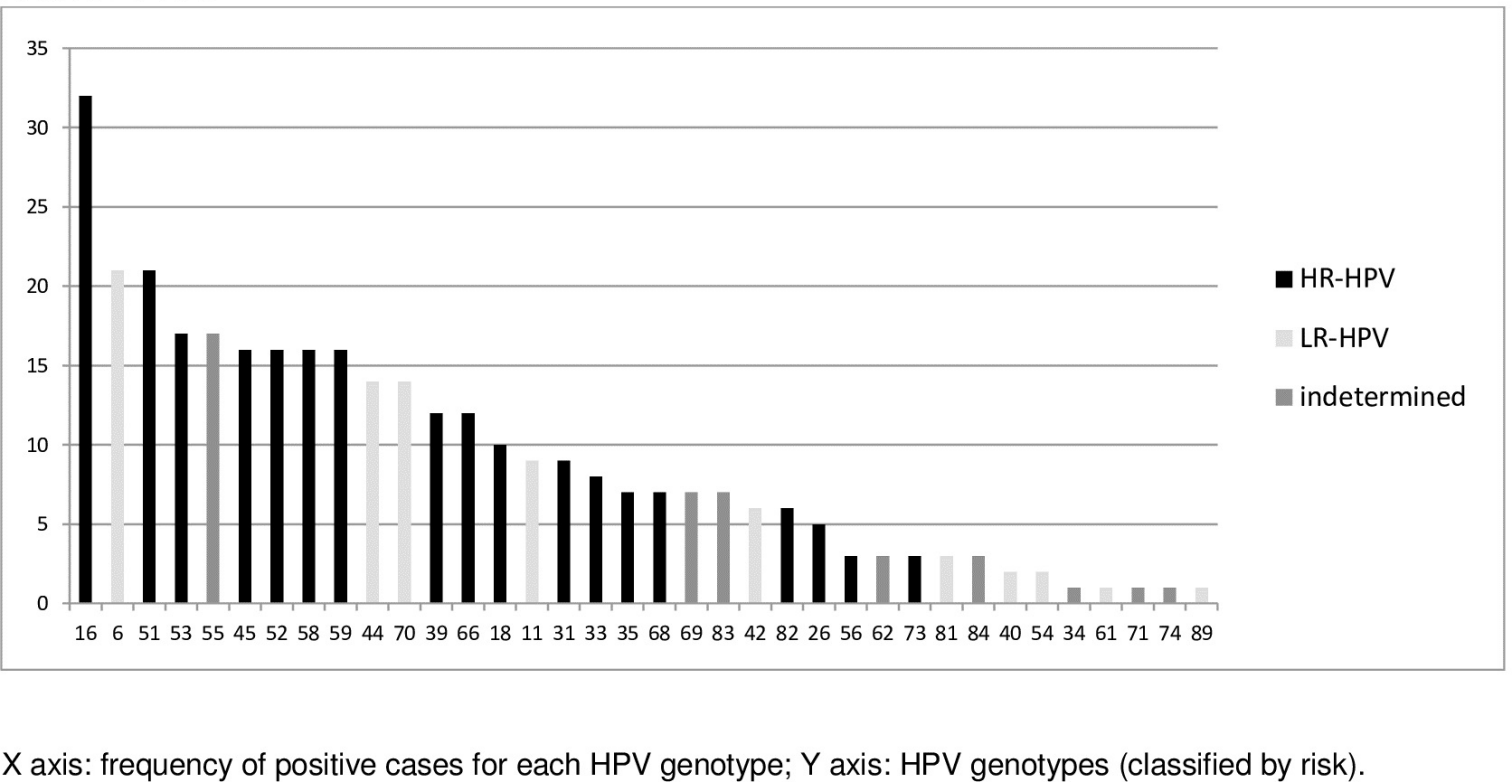
Distribution of HPV genotypes in HIV patients attending a STI clinic in Vitoria, Brazil (n = 223).

HR-HPV was found in 50.7% (113/223) of the total samples and 74% (113/153) of sample when considering only the HPV-positive cases. HPV16/18 cases were very frequent (40.7%; 46/113), in 32% of HR-HPV positive cases in men and 67.4% in women. The frequency of other HPV genotypes is shown (Fig 1). The URR sequencing was successfully carried out in 38 samples identified as HPV16-positive, and European variants were the most frequent (69.2%), with similar frequency in both groups, followed by African (25.6%) and Asiatic-Americans (5.1%).

Behavioral data and their association with HPV are shown in (Table 2). Being younger than 35 years of age was associated to any HPV type (p = 0.002) and multiple HPV infection (p = 0.001); having more than 20 sexual partners was associated with multiple HPV infection (p = 0.000); anal sex was associated with the presence of any HPV (p = 0.001). Reporting of a first intercourse before 15 years of age was linked to any HPV (p = 0.000) and HR-HPV (p = 0.004). Being MSM (Men who have sex with men) was a risk factor for any HPV (p = 0.004), HPV16/18 (p = 0.041) and multiple infections (p = 0.003).

**Table 2:** Behavioral aspects of HIV patients attending an STI clinic in Vitoria, Brazil (n = 223).

Variable	Any HPV (n = 153)	HR-HPV (n = 113)	HPV16/18 (n = 46)	Multiple HPV types (n = 85)
**Age**				
< 25 years	12 (92.3)	09 (69.2)	04 (30.8)	06 (54.5)
26–35 years	42 (80.8)	32 (61.5)	12 (23.1)	29 (55.8)
36–50 years	79 (63.2)	60 (48.0)	25 (20.0)	41 (33.1)
> 50 years	20 (60.6)	12 (36.4)	05 (15.2)	09 (27.3
X^2^	9.640	10.731	1.642	11.033
*p-value*	0.022	0.097	0.650	0.012
**Number of Partners**				
< 5 partners	34 (57.6)	27 (45.8)	14 (23.7)	16 (27.1)
6–20 partners	59 (70.2)	39 (45.9)	13 (15.3)	27 (32.1)
21–50 partners	32 (82.1)	24 (61.5)	07 (17.9)	22 (57.9)
> 50 partners	28 (70.0)	23 (57.5)	12 (30.0)	20 (51.3)
X^2^	6.637	7.274	4.140	13.370
*p-value*	0.013	0.014	0.058	0.000
**Wearing condoms**				
Never	31 (75.6)	28 (68.3)	13 (31.7)	15 (36.6)
Sometimes	42 (66.7)	28 (44.4)	05 (7.9)	29 (46.0)
Always	80 (67.2)	57 (47,9)	28 (23.5)	41 (35.3)
X^2^	1.149	7.039	9.884	2.056
*p-value*	0.563	0.134	0.070	0.358
**Anal sex**				
Yes	122 (74.8)	92 (56.4)	39 (23.9)	71 (44.1)
No	31 (51.7)	21 (35.0)	07 (11.7)	14 (23.7)
X^2^	10.942	8.247	4.026	7.557
*p-value*	0.001	0.016	0.045	0.006
**First sexual intercourse before 15 years of age**				
Yes	60 (84.5)	47 (66.2)	14 (19.7)	33 (47.8)
No	93 (61.2)	66 (43.4)	32 (21.1)	52 (34.4)
X^2^	12.223	11.569	0.053	3.581
*p-value*	<0.0001	0.003	0.819	0.058
**Drug use**				
Yes	46 (68.7)	36 (53.7)	15 (22.4)	33 (49.3)
No	107 (68.6)	77 (49.4)	31 (19.9)	52 (34.0)
X^2^	0.00	3.183	0.181	4.581
*p-value*	0.992	0.204	0.670	0.320
**Smoking**				
Yes	30 (66.7)	25 (55.6)	11 (24.4)	21 (46.7)
No	123 (69.1)	88 (49.4)	35 (19.7)	64 (36.6)
X^2^	0.084	0.608	0.502	1.539
*p-value*	0.450	0.738	0.479	0.215
**Sexual identity**				
Woman	96 (67.1)	70 (49.0)	31 (21.7)	49 (34.5)
MSM	48 (81.4)	37 (62.7)	15 (25.4)	32 (56.1)
Straight man	09 (42.9)	06 (28.6)	0 (0)	04 (19.0)
X^2^	11.062	8.005	6.383	11.786
*p-value*	0.004	0.091	0.041	0.003

n: number of people within the population; Any HPV: Any HPV; HR-HPV: high-risk HPV; HPV 16/18: HPV 16 or 18; Multiple HPV: more than one type of HPV in the same person; MSM: men who have sex with men.

HPV infection and HIV data are shown in (Table 3). CD4 count >500cells/mmᶟ was a protective factor for HPV16/18 (p = 0.048) and multiple infections (p = 0.023); the undetectable viral load was protective for any HPV (p = 0.046), HR-HPV (p = 0.003) and multiple infections (p<0.001), and not being in a program of HAART treatment was a risk factor for any HPV (p = 0.010) and multiple infections (p = 0.006). Abnormal anoscopy was found in 23.7% of the total patients (53/223), and it was significantly associated with all types of investigated HPV infections.

**Table 3:** Prevalence and correlates associated with anal HPV infection in HIV patients attending an STI clinic in Vitoria, Brazil (n = 223).

Variable	Any HPV (n = 153)	HR-HPV (n = 113)	HPV16/18 (n = 46)	Multiple HPV types (n = 85)
**HIV time**				
Less than 5 years	69 (75.0)	52 (56.5)	20 (21.7)	42 (46.7)
5–10 years	36 (57.1)	25 (39.7)	08 (12.7)	17 (27.0)
11–20 years	45 (70.8)	35 (53.8)	17 (26.8)	24(37.5)
Over 20 years	03 (100.0)	01 (33.3)	01 (33.3)	02 (66.7)
X^2^	6.975	7.349	3.997	7.085
*p-value*	0.073	0.060	0.262	0.069
**Undetectable viral load**				
Yes	86 (64.2)	56 (41.8)	20 (14.9)	36 (27.1)
No	64 (77.1)	54 (65.1)	23 (27.7)	47 (58.0)
X^2^	3.998	11.396	5.273	20.320
*p-value*	0.046	0.003	0.220	<0.0001
**CD4 count (cells/mmᶟ)**				
< 200	12 (80.0)	10 (66.7)	06 (40.0)	10 (66.7)
200 a 349	23 (76.7)	16 (53.3)	09 (30.0)	13 (44.8)
350 a 500	30 (76.9)	25 (64.1)	04 (10.3)	18 (47.4)
> 500	85 (62.5)	59 (43.4)	25 (18.4)	43 (31.9)
X^2^	6.314	11.577	12.278	11.479
*p-value*	0.277	0.314	0.031	0.043
**HAART**				
Yes	117 (64.6)	85(47.0)	36 (19.9)	61 (34.1)
No	35 (85.4)	27 (65.0)	09 (22.0)	23 (57.5)
X^2^	6.651	4.785	0.880	7.585
*p-value*	0.010	0.091	0.767	0.006
**Previous STI**				
Yes	100 (69.4)	73 (50.7)	35 (24.3)	59 (41.8)
No	44 (64,7)	39 (50.0)	10 (14.7)	19 (27.9)
X^2^	0.811	0.113	2.546	3.791
*p-value*	0.368	0.945	0.111	0.052
**Abnormal anoscopy**				
Yes	32 (97.0)	23(69.7)	14 (42.4)	19 (57.6)
No	57 (45.2)	42 (33.3)	14 (11.1)	29 (23.0)
X^2^	28.399	14.595	17.673	14.820
*p-value*	<0.0001	0.0001	<0.0001	0.0001
**Abnormal anal cytology**				
Yes	41 (77.4)	32 (60.4)	18 (34.0)	28 (54.9)
No	103 (66.5)	72 (46.5)	35 (14.8)	51 (33.1)
X^2^	2.206	3.202	9.127	7.677
*p-value*	0.138	0.202	0.003	0.006
**AIDS-defining illness**				
Yes	38 (76.0)	32 (64.0)	10 (20.9)	21 (42.0)
No	115 (66.9)	81 (47.1)	36 (16.7)	64 (37.0)
X^2^	1.511	5.054	0.200	0.277
*p-value*	0.219	0.080	0.886	0.599

n: number of people within the population; Any HPV: Any type of HPV; HR-HPV: high-risk HPV; HPV 16/18: HPV 16 or 18; Multiple HPV: more than one type of HPV in the same person; HAART: highly active antiretroviral therapy, CD4: CD4 + T lymphocytes rate per microliter blood.

The comparison of behavioral aspects of anal cytology results are shown. (Table 4). Fifty-three (25.5%) participants were diagnosed with abnormal cytology.

**Table 4:** Comparison of behavioral aspects by anal cytology results in HIV patients attending an STI clinic in Vitoria, Brazil (n = 223).

Variable	Abnormal cytology (n = 53)	Normal cytology (n = 155)
**Number of partners**	**n (%)**	**n (%)**
< 20	29 (21.2)	107 (75.9)
> 20	24 (33.8)	47 (66.2)
X^2^	3.814	3.814
*p-value*	0.051	0.051
**Wearing condom**		
Never	07 (20.0)	31 (81.6)
Sometimes	13 (24.5)	46 (78.05)
Always	33 (34.0)	78 (70.3)
X^2^	2.422	2.422
*p-value*	0.298	0.298
**Anal sex**		
Yes	45 (21.6)	108 (51.9)
No	8 (4.0)	47 (22.6)
X^2^	4.709	4.709
*p-value*	0.030	0.030
**First intercourse <15 years of age**		
Yes	20 (29.4)	48 (70.6)
No	33 (23.6)	107 (76.4)
X^2^	0.822	0.822
*p-value*	0.365	0.365
**Use of drugs**		
Yes	24 (38.1)	39 (61.9)
No	29 (20.0)	116 (80.0)
X^2^	7.573	7.573
*p-value*	0.006	0.006
**Smoking**		
Yes	14 (26.4)	29 (55.6)
No	39 (23.6)	126 (48.0)
X^2^	1.430	1.596
*p-value*	0.232	0.206
**Sexual identity**		
Woman	26 (19.4)	108 (80.6)
MSM	24 (43.6)	31 (56.4)
Straight men	03 (15.8)	16 (84.2)
X^2^	13.094	13.094
*p-value*	0.001	0.001

MSM: men who have sex with men.

## Discussion

This study is the first epidemiological study conducted in Espírito Santo state, Brazil, that reports the prevalence rates, the distribution of HPV genotypes and the factors associated with anal HPV infection in HIV patients. The prevalence of anal HPV infection was frequent in this study (68.6%), and it was similar in both genders, even when considering HR-HPV and HPV16/18 infections. In the study population, MSM presented a significantly higher prevalence of HPV, as did having more than 20 partners. This result is similar to results of other studies that were conducted worldwide [7,8], and it points out the role of risk behavior in STI infections. Also, in this study, a first sexual intercourse before 15 years of age has a strong correlation with the HR-HPV infection.

Regarding the HPV genotypes, the tests used were able to identify 39 different types of HPV. The most frequent HR-HPV types were 16, 51 and 52, and the most frequent LR-HPV types were 6, 53 and 70. Some of the types have a low prevalence in the general population but present higher frequency in HIV population [8,30]. The HPV16 was the genotype that was more frequently found in our samples, and together with the HPV18 was significantly associated with abnormal anoscopy and abnormal anal cytology. We expect that following the HPV vaccination in young people, after a period of a few years, there should be a decrease the intraepithelial lesions caused by HPV 16 within a general population, this should also be true for cases of also HIV-seropositive [14; 31].

The results of a global review concluded that 84% of the invasive anal cancer cases contain HPV DNA, most individuals (87% of those who were HPV-positive) were positive for HPV16, and fewer (6%) were positive for HPV18 [32]. In a meta-analysis study, HPV was very common in the anal canal, and the causative agent of most anal cancer, of which HPV16 was detected in about one third (35%) of the HIV-positive men but only about one in eight (13%) of HIV-negative men [23]. The association of HPV16 with carcinoma could be explained by its elevate persistence rate which is higher than other HR-HPV types [33,34], and some HPV16 variants also showed a reduced clearance frequency [35,36]. Our study showed a higher frequency of HPV16 European variants and no association between non-European variants and AIN; the predominance of European variants in this study implies a lower risk for the development of HSIL as previously described in the body of literature covering our region [37]. Investigating HIV-negative patients in the same geographic area [38], found a consistent association between HPV16 non-European variants and high-grade cervical intraepithelial lesions [8]. This fact raises the relevance in studying the relation between variants and anal lesions since this relation is well documented for cervical malignancy [35,36,37].

In our study several socio-demographic characteristics had significant differences between men and women; higher education was more frequent in men; the more recent HIV diagnoses (less than 5 years) was more frequent in men, even with an average age over 40 years. Regarding clinical variables, HIV viral load and low CD4 counts were significantly associated with HR-HPV and multiple infections, which could raise the risk of persistent infection and consequently, of malignant development. Our result showed that HAART therapy was protective for multiple HPV infection, and it was previously demonstrated that HR-HPV prevalence was lower in patients in the long-term application of HAART [39, 40]. However, the incidence of HPV-induced lesions in the anal canal are still high in patients on a program of HAART [41], and the frequency of anal cancer was no different in the pre-HAART and HAART eras [42]. These results could suggest that the immunological restoration by medication was not enough for the clearance of HPV-persistent infection.

The prevalence of abnormal anal cytology (25.5%) shows that it is a common finding in HIV-infected patients. Repeated anal cytology testing and referral for HRA could increase the likelihood of AIN diagnosis. The highest positivity of abnormal anal cytology in our study occurred in the MSM population (43.6%), wich is similar to the rate of 53.6% described in a Spanish cohort of MSM that was HIV-infected [43]. Our study found one case of anal adenocarcinoma and two cases of AIN II in HIV positive women; however, because the disease prevalence is much higher in HIV-infected MSM compared with women, it is necessary to undertaken further studies to define the best screening tool for HIV positive women. We had a high concordance of HRA with positive biopsies (90.6%), showing that HRA is an important test for increasing the diagnosis of AIN, a similar result to the study by Gimenez in Manaus in Amazonas state, Brazil, which found a high sensitivity of the HRA, 90.0% [27]. In our study, 18.3% of HRA were positive for 159 examinations executed, a higher rate of positivity in MSM (41.4%). In general, AIN prevalence rates range from 10.4% to 68.0% among asymptomatic patients (28–30). The gold standard diagnostic for AIN is histopathology. For now, it is possible to note that anal cytology may be a screening test for AIN and cancer in an HIV population.

We found some study limitations, for example, missing data regarding additional tests such as anal cytology due to unsatisfactory samples, although all patients underwent anal cytology. Some patients refused to undergo anoscopy due to complaining of pain or bleeding. We did not have an additional blinded pathologist to perform confirmation of the results. Due to the low frequency of reporting some risk factors in the samples, the number of subjects studied was sufficient enough to find a statistical association between some independent variables and anal HPV infection. The possibility of having been response bias cannot be ruled out due to the general tendency to give socially acceptable answers. However, the limitations do not diminish the importance of this study in providing visibility to a neglected population during preventive actions in assisting HIV-infected individuals.

Some aspects of both genital cancers (cervical and anal) are shared, as the HPV infection and persistence as a risk factor, HPV16 as the main associated genotype and progression to carcinoma after the emergence of the previous intraepithelial lesions.

Since the HR-HPV prevalence and the frequency of the related cancers are strictly associated and have a higher risk in HIV positive individuals, we believe the introduction of HPV DNA testing in this population could have an important role in stratifying the risk of HIV-seropositive patients, as has been proposed by other researchers in the literature [44].

## Conclusions

These results reinforce the need to establish an anal cancer screening on high-risk patients, such as HIV-positive women and men. The anus and cervix susceptibility to HPV infection and these HPV-induced malignancies might be explained by the same embryological origins. Anal and cervical cancers have similar tumor biology; hence a similar cancer screening program would appear to be reasonable, considering the success of cervical cancer screening programs around the world [45]. A co-test using anal cytology and HPV genotyping may be considered a useful tool, especially considering the increase in sensitivity when both tests are used [46]. Patients at risk of anal cancer, such as those who are HIV-positive, especially with the lower CD4 count, and MSM, would benefit with an earlier diagnosis if we considered the identification of HR-HPV infection before the emergence of the abnormal cells [47].

Currently, the Centre for Disease Control and Prevention (CDC) in the USA recommends that high-risk populations be vaccinated using the quadrivalent or nonavalent vaccines [22]. In Brazil, their recommendations for vaccination against HPV infection include immunocompromised, MSM and HIV patients in a broad age range [23]. This proposal shows the concern regarding HPV infections and consequent development of lesions within the risk groups, hence we believe that anal screening, improved by molecular tests, in the HIV population will be the next step toward reducing HPV-related morbidity and mortality [48].

## Supporting information

S1 File Questionnaire. (DOCX)

S2 File Data base. (SAV)
