# Supplementary material for: Anal HPV infection and correlates in HIV-infected patients attending a Sexually Transmitted Infection clinic in Brazil
Source: PLoS One. 2018 Jul 5;13(7):e0199058. doi: 10.1371/journal.pone.0199058 (PMC6033400; doi:10.1371/journal.pone.0199058)
Supplement: S1 File — (DOCX) [file pone.0199058.s001.docx]

**Questionnaire**

Initial data:

Number of records:

District / Town of Residence:

- Naturalness:
- Birthdate:
- Age? | __ | __ | years
- Scholarity (in years): __________________ Sexual Option: --------------------------
- **What is your marital status?**

Single 2. Married / Living together Marital status 3. Separated 4. Widowed

- **Occupation: ____________________________**
- **Family income:**

1. Up to 1 minimum wage 2. From 1 to 3 SM 3. From 3.1 to 5 SM 4. More than 5

- **How many people live in your house**? _______________________

**USE OF CIGARETTES AND DRUGS**

- **You smoke?**

1. Yes, How many cigarettes?

A.Irregularly, from time to time B. Less than 5 cigarettes per day

C.From 5 to 20 cigarettes per day D. More than 20 cigarettes per day

2. No

If not, have you ever smoked?

A. Yes, 1 or 2 times B. Yes, regular C. No, never

- **For a week or so, have you had any alcohol?**

1.No 2. Once 3. Often 4. Became drunk

- **Do you serve any type of medication?**

1.For anxiety and stress 2. Antidepressants 3.No 4.TARV If so, which one?_________

- **Do you already use some kind of drug?**

1.No 2. Shoe repair glue, ether 3. Cocaine 4. Crac 5. Legal drugs 6.Other ___________

- **Have you ever used the injectable drug?**

1.Yes 2. No 3. NR

**CONTRACEPTIVE INFORMATION**

- **Do you use any contraceptive method?**

1.Pilula 2. Condom 3. IUD 4. Cervical mucus/Billing 5. Calendar-based method 6. Basal temperature 7. Diaphragm 8. Next day pillow 9. Female condom 10. No

- **Do you and your partner use condoms?**

1. Yes 2. No

- **If yes, how often do they use condoms?**

1. Always 2. Sometimes 3. Rarely 4. Never

**SEXUAL AND REPRODUCTIVE HISTORY**

- **How old were you when you first had intercourse? | __ | __ | years**
- **How old was your partner? | __ | __ | years**
- **Have you ever had anal intercourse**?

1. Yes 2. No 3. NR

- **Have you ever had sex with a person of the same sex?**

1. Yes 2. No 3. NR

- **On average, how many sexual intercourses do you have per week?** | __ | __ | __ |
- **How many sexual partners did you have in your life?** | __ | __ | __ |
- **How many sexual partners did you have in the last 12 months? | __ | __ | __ |**
- **Have you ever received money for having sex?**

1. Yes 2. No 3. NS

- **Have you ever had sexual intercourse against your will?**

1.Yes 2. No 3. NR

- **If so, at what age? | __ | __ | Was this repeated?**

1.Yes 2. No 3. NR

- **If so, how many times? | __ | __ | __ |**
- **Who was a person**?

1.Someone in your family, 2. an adult 3. Someone in your family, a young person

4.One known 5. One unknown 6. Several people

- **You've got pregnant already**?

1.Never 2. Once 3. Several times 4. Not applicable

- **If so, what was your age at first pregnancy?** | __ | __ | years

**How many children ( ) Spontaneous abortions ( ) Spontaneous abortions ( )**

- **Are you pregnant?**

1. yes 2. no 3. does not apply

**HEALTH, STD, AND AIDS**

- **When was the last time you took a gynecological (preventive) exam? ______**
- **Do you know that she has ever had a sexually transmitted disease?**

1. Yes 2. No

- **If so, which one? __________________-**
- **How did you know this disease/infection?**

1. Your partner said that you were infected? 2. Have you received symptoms that led to a health service? 3. During a routine visit or other reason.

- **At the time of the occurrence, you sought**

1.No one 2. A doctor / Family health program 3. A drugstore 4. STD Clinic

- **Do you have a partner that you had an STD?**

1. Yes 2. No

- **After the diagnosis of STD have you used the condom more often?**

1.Yes 2. No

- **After the diagnosis of STD, do you reduce your sexual activity?**

1. Yes 2. No

- **You can find out about your partner:**

1. Have other partners 2. Use drugs 3. Already been prisoner

4.History of STD 5. Is HIV positive 6. Other _____________________

- **Have you been tested for HIV**?

1. On its own initiative 2. During a hospitalization 3. During prenatal care 4. During DST clinic care 5. In other circumstances

- **Do you have any of the symptoms?**

1. Pelvic pain 2. Frequent genital bleeding 3. Itching 4. Lacerations 5. Inguinal adenopathy (thigh groin) 6. Burning while urinating 7. Genital wound 8. Other:

**TESTS RESULTS:**

- Anal cytology:
- Anoscopy:
- Biopsy:
- HPV test:
- Variants:
- CD4:
- Viral charge:
- Comments:
